# What will it take? Perspectives from five low- and middle-income countries on opportunities and challenges of introducing new maternal vaccines

**DOI:** 10.1016/j.vaccine.2024.126654

**Published:** 2025-01-25

**Authors:** Ranju Baral, Jessica A. Fleming, Iracema Barros, Patience Cofie, Patience Dapaah, Sadaf Khan, Sophia J. Knudson, Sandeep Kumar, Kamran Mehedi, Patrick K. Munywoki, Lauren Newhouse, Bryan O. Nyawanda, Joyce U. Nyiro, Joseph Odiyo, Elkanah Otiang, Rosemond Owusu, Clint Pecenka, Melanie Picolo, Judite Pinto, Diana Quelhas, Satyabrata Routray, Sarah Sultana, Surendra Uranw

**Affiliations:** a PATH, Seattle, United States of America; b PATH, Maputo, Mozambique; c PATH, Accra, Ghana; d PATH, New Delhi, India; e PATH, Dhaka, Bangladesh; f Kenya Medical Research Institute - Wellcome Trust Research Programme (KEMRI - WTRP), Kilifi, Kenya; g Kenya Medical Research Institute - Center for Global Health Research (KEMRI - CGHR), Kisumu, Kenya; h Independent Consultant, Nairobi, Kenya; i PATH, Nairobi, Kenya; j Independent Consultant, Accra, Ghana; k Independent Consultant, Maputo, Mozambique; l Independent Consultant, Dhaka, Bangladesh; m B.P. Koirala Institute of Health Sciences (BPKIHS), Dharan, Nepal

**Keywords:** Maternal immunisation, Vaccine implementation, Low-and middle-income countries, Respiratory syncytial virus, Group B streptococcus

## Abstract

**Background:**

New vaccines for pregnant women have recently been introduced in some high-income countries to protect infants in early life. Implementing maternal immunisation (MI) successfully in low- and middle-income countries will require planning and adaptations to immunisation and maternal health programs. To inform cost of MI delivery studies, we gathered perspectives from key stakeholders in five countries (Bangladesh, Ghana, Kenya, Mozambique, and Nepal) regarding health system requirements, opportunities, and challenges to introducing new maternal vaccines into routine health programs.

**Methods:**

We convened national stakeholders for country workshops to deliberate potential delivery strategies, opportunities, and constraints for implementing new MI interventions. Divided into thematic areas, participants discussed health system adaptations needed to establish or strengthen an MI platform.

**Findings:**

Stakeholders felt that existing programs that administer vaccines to pregnant women, primarily tetanus-containing vaccines, provide a proven path for the delivery of additional maternal vaccines. Maternal health and immunisation program integration was perceived strongest at the health care provision level and weakest at the national level. Concerted coordination at the national level was identified as critical to the success of introducing new interventions. Stakeholders perceived that most health workforce providing immunisation and antenatal care services undergo similar training and have transferrable skills, however developing operational guidelines with clear roles and responsibilities was deemed critical for ownership and accountability. Human resource constraints were cited as a challenge for some countries. The importance of raising awareness of disease burden and new MI interventions was also highlighted across countries. Sustainable financing was a recurring challenge cited, especially with transitions from Gavi support.

**Conclusions:**

The workshops enabled country stakeholders to deliberate needs to support the introduction of new maternal vaccines. While circumstances and needs differed, stakeholders across countries were optimistic about the capability of their health systems to integrate new maternal vaccines.

## 1 Introduction

Maternal immunisation (MI), or vaccination during pregnancy, is a strategy for preventing infectious diseases in mothers and/or infants during the earliest months after birth [1]. A maternal vaccine against the leading cause of pneumonia in infants and young children, respiratory syncytial virus (RSV), has recently been licensed [2], and other maternal vaccines are in development (e.g., Group B *Streptococcus* [GBS]) [3]. With the recent policy recommendation from the World Health Organization's Strategic Advisory Group of Experts on Immunisation, maternal RSV vaccines are expected to be available for use in low- and middle-income countries (LMICs) as early as 2025 [4]. Understanding the potential delivery strategies of these products is important for informed decision-making, especially in LMICs, where affordability and sustainability of vaccination programs are questionable as countries transition away from Gavi, the Vaccine Alliance vaccine financing support [5,6].

Currently, vaccines provided to pregnant women, such as tetanus toxoid (TT) or tetanus diphtheria (Td), are often delivered through the Expanded Programme on Immunisation (EPI) system, leveraging parts of the antenatal care (ANC) platform [5]. However, introducing novel maternal vaccines through existing health systems may require adaptations to immunisation and maternal health programs to optimize their delivery [6]. As circumstances and needs will likely vary between countries, individuals with local experience and knowledge can provide context-specific insight into country capacity and strengths and limitations of respective health systems. We report here on perspectives from stakeholder workshops conducted in five LMICs (Bangladesh, Ghana, Kenya, Mozambique, and Nepal) to identify health system requirements, opportunities, and challenges to introducing new maternal vaccines into their routine health programs.

## 2 Methods

PATH, an international, non-government organization (NGO), hosted five country stakeholder workshops in collaboration with respective ministries of health (MOH) in Bangladesh, Kenya, Mozambique, and Nepal and the Ghana Health Service between June 2022 and March 2023. The Kenya Medical Research Institute and B.P. Koirala Institute of Health Sciences co-hosted in Kenya and Nepal, respectively (Table 1). The workshops, conducted to inform MI cost of delivery studies in each country, invited stakeholders to deliberate feasible delivery strategies for successful implementation of forthcoming MI interventions; groups ranged between 36 and 57 individuals (Supplementary material 1). Participants were selected based on their subject matter expertise and professional positions in relevant institutions or decision-making bodies in each country, based on recommendations and referrals from the study team and EPI and maternal child health (MCH) program managers. Participants included policymakers, public health practitioners, national and sub-national MOH representatives from immunisation and MCH departments, academics, national immunisation technical advisory group members, and representatives from professional associations, research organizations, NGOs, and civil society organizations (Supplementary material1).

**Table 1:** Country stakeholder workshops details.

Countries	Co-hosts	Workshop date	Number of participants
Kenya	MOH^1^, PATH, KEMRI^2^	15 June 2022	44
Ghana	MOH^1^, GHS^3^, PATH	1 February 2023	46
Bangladesh	MOH^1^, PATH	12 March 2023	57
Nepal	MOH^1^, PATH, BPKIHS^4^	16 April 2023	37
Mozambique	MOH^1^, PATH	24 May 2023	35

1 Ministry of Health; ^2^Kenya Medical Research Institute; ^3^Ghana Health Service; ^4^B.P. Koirala Institute of Health Sciences.

The workshop agendas were organized into three sessions. First, background information was shared in plenary on MI and upcoming interventions with a focus on vaccines against RSV and GBS, target diseases, and their global and local burden. Next, MOH representatives summarized their country's health care delivery system and regulatory processes specific to immunisation and MCH in plenary. These presentations highlighted current delivery mechanisms and programmatic issues related to maternal tetanus immunisation and ANC provision.

Finally, participants self-selected into groups based on their expertise and interest to discuss one of six topics: 1) procurement, logistics, and supply chain management; 2) program planning and management; 3) training and supervision; 4) vaccine administration and surveillance; 5) monitoring, evaluation, and information system management; and 6) demand creation, including sensitization, social mobilization, and communication. In each group, comprised of four to eight participants, a chairperson guided discussions using the following questions and additional prompts (Supplementary material 2):

1. How would the thematic area need to change to support a platform of MI interventions?
2. What new or additional thematic area would be needed to support an MI platform of interventions, if any?
3. How would roles, responsibilities, and interactions work or need to change across EPI and ANC programs at different levels of the health system?
4. What are the biggest opportunities and challenges/barriers anticipated around the MI intervention thematic area?

Scribes took notes and a rapporteur for each group provided summaries in a presentation in the final plenary session. Reporting was predominantly conducted in English. We reviewed written notes and presentations and deductively and inductively identified key topics, using frequency scores to identify key themes. We did not attribute sentiments to specific individuals and weighted all responses equally. We present pooled findings and highlight country-specific variations.

## 3 Findings

Key themes recurred across the groups in all five countries. These are summarized in Table 2 and elaborated upon below.

**Table 2:** Key themes emerging from workshops across countries.

Maternal immunisation (MI) delivery mechanisms	Integration and workforce	Awareness and demand	Financing and sustainability
Current systems can be utilized for implementing new MI. • Current mechanisms for delivering MI can support the implementation of new MI interventions. • The roles of maternal, child health (MCH) and Expanded Programme on Immunisation (EPI) programs vary by country.	Integrated service delivery is implemented at some health facility levels but not all. • Integration between MCH programs and EPI is most robust at the health care provision level but least effective at the national level. • Achieving integrated delivery of new MI interventions will necessitate health workers taking ownership and accountability. • Health worker shortages pose challenges to the introduction of new vaccines.	Increased awareness is needed regarding target disease burdens and new MI interventions. • Raising awareness among health workers and communities will be crucial for successfully introducing new maternal vaccines. • Addressing community concerns and information needs regarding maternal vaccines will be important.	Financial limitations and competing priorities pose significant barriers to introduction. • Introduction decisions and program sustainability will rely on the availability of financing from entities like Gavi, The Vaccine Alliance and other forms of external support. • Most countries are encountering economic challenges, including budget cuts in financing current routine EPI vaccines.

### 3.1 MI delivery mechanisms

Participants across all countries recognized and valued the long experience of vaccinating pregnant women with TT-containing vaccines and expected that the existing delivery mechanisms could support new MI interventions, though they also acknowledged constraints with current systems that may affect new MI administration.

Current models for MI delivery (i.e., TT or Td), including roles of MCH and EPI programs, vary by country and level of health care. In Ghana, midwives generally administer vaccines during routine ANC visits, whereas in Kenya and Mozambique, pregnant women receive vaccines during ANC visits from midwives or immunisation program staff. In Bangladesh and Nepal, pregnant women are mostly referred to EPI sessions for vaccination (held once or twice monthly), necessitating additional visits outside routine ANC appointments.

Participants in Bangladesh noted a wide urban-rural disparity in health care delivery, including large hard-to-reach urban populations. Primary health care in these urban settings is often managed by NGOs and the private sector; participants recommended strengthening urban delivery structures, including robust immunisation tracking and monitoring systems. Participants from Kenya also noted the importance of the private sector in immunisation and in shortening the time to vaccine availability.

Health care workers providing immunisation and ANC services typically have similar foundational basic training with transferable skills across all countries. Notably, in Kenya, staff rotate between ANC and EPI roles every few weeks and ANC and EPI services are frequently offered in the same facility, presenting opportunities for resource integration. Participants from all countries cited a preference for a “one-stop shop” where pregnant women can conveniently access vaccines to enhance service coverage. For high-volume ANC settings, however, referring pregnant women to EPI for vaccination was cited as more practical in Nepal and Mozambique.

Unlike TT vaccination, which can be administered at any time during pregnancy, new maternal vaccines are recommended at specific gestational ages for optimal effectiveness [4], [7]. Participants in Kenya, Nepal, and Mozambique, did not anticipate significant challenges in identifying and reaching target populations within the timeframe of 24-36 weeks, citing perceptions that most pregnant women attend ANC visits in late second or early third trimester of pregnancy, which are more likely to coincide with the recommended vaccination window for RSV maternal vaccine.

### 3.2 Integration and workforce

Participants across all countries viewed MI interventions as a bridge between immunisation and MCH services and highlighted the importance of integrating relevant policies and services between the EPI and MCH programs for successful implementation. While degrees of integration across the programs vary between countries, participants from all countries except Nepal generally perceived coordination as strongest at the delivery level and weakest at the national level. Participants across all countries highlighted the importance of having operational guidelines with clearly delineated roles and responsibilities for fostering ownership and accountability across departments and levels of implementation.

The consensus among workshops participants across all five countries was that including MCH experts in MI policymaking bodies was important. Establishing their representation in decision-making committees at the national level was particularly emphasized in Ghana.

Across all countries, EPI programs generally handle procurement, logistics, and supply chain management for immunisation, a structure that participants favored for future MI interventions. Enhancing functional integration in other service delivery areas through improved coordination between departments, however, was a recognized need across all countries.

Health worker shortages were cited as significant barriers to MI implementation in some countries (Bangladesh, Ghana, and Kenya), especially among midwives that administer vaccines to pregnant women amidst already strained resources. Participants from Bangladesh, for instance, cited challenges associated with high vacancy rates in vaccinator positions. Although not specific to MI, this shortage remains a critical constraint.

Logistics management was cited as crucial for successful introduction of new interventions across all countries. Compatibility with existing cold chain requirements was specifically noted as a key consideration for introducing new vaccine products in Ghana and Kenya. Products that align with standard cold chain protocols and specifications were perceived as easier to integrate into existing logistics systems. Participants also highlighted the need to evaluate system capacity for vaccine storage, distribution, monitoring, reporting, and information management before vaccine introduction.

Despite advances in digital health information systems across countries, participants in Kenya, Ghana, and Bangladesh cited challenges with recording and reporting vaccination coverage, particularly tetanus vaccine. To improve accuracy and efficiency in reporting, participants recommended adapting digital systems for MI and implementing unique patient identification numbers to facilitate data sharing between EPI and MCH programs.

### 3.3 Awareness and demand

Understanding the epidemiology and burden of MI target diseases could help prioritize prevention interventions in national policies. While participants in all countries cited low awareness of the burden of RSV and GBS and new MI interventions, consensus was strong that greater awareness was needed. Opportunities to increase awareness across all countries included adding information on MI target diseases and prevention tools to basic training packages and continuing medical education for health workers and creating job aids for service providers across all levels of care. While a concern cited in Kenya was raised about over-burdening health workers by adding another task to their responsibilities, participants also acknowledge that over the long term, health workers' time could be saved if maternal vaccination significantly reduced the burden of childhood disease.

Participants across all countries cited community education as important and recommended leveraging existing communications and social mobilization tools to raise awareness of disease targets and upcoming interventions as well as improve vaccine confidence and address vaccine hesitancy, which was widely cited as a growing concern. Ideas to increase community awareness included displaying advertisements on clinic televisions, sending information through mobile phones to key audiences, and adding MI-specific content to routine health talks and outreach activities. Identifying and engaging spokespersons and champions to support knowledge and information sharing around MI was also cited as important in all countries.

Incentive programs exist in Nepal and Bangladesh to encourage pregnant women to receive maternal health services, including ANC, and institutional delivery. Participants in Nepal suggested that MI could be added to the maternal and child health package for which women would receive incentive benefits.

### 3.4 Financing and sustainability

Participants from all countries identified the importance of sustainable financing in MI introduction decisions, especially considering country transitions away from immunisation support from Gavi (Table 3). Across all countries, a concern among participants was that health programs are often driven by global priorities. Financial downturns and competing priorities were highlighted as major introduction barriers for new maternal vaccines and even for the sustainability of current routine EPI vaccines. Participants in Ghana asked about the availability of combination instead of single antigen vaccines as one way to reduce implementation costs of new maternal vaccines. Discussions suggested that the availability of combination vaccines would likely be favored by countries as the number of vaccines in the schedule continue to expand. Stakeholders in Ghana emphasized the need for investment at the global level for the development of combination vaccines. Sharing information on disease burden, product characteristics, efficacy, and costs were identified as important inputs in prioritizing interventions across all countries. Participants in Mozambique highlighted the importance of ensuring a sustainable vaccine supply before considering vaccine introduction, citing recent experiences of inadequate supply that resulted in program interruptions. Participants from Kenya also highlighted the vital role that the private sector plays in immunisation and noted Typhoid and human papillomavirus vaccines were available in the private sector long before government facilities. The private sector should, therefore, be included as important partners for MI sustainability.

**Table 3:** Transition status from Gavi, The Vaccine Alliance support.

Country	Funding Phase	Funding phase definition
Bangladesh Ghana Kenya	Accelerated transition (Phase 3)	Countries within gross national income per capita above the US$1730 threshold when its three-year average gross national income as well as its most recent gross national income are above the eligibility threshold, and the country is co-financing at least 35 % of vaccine costs

Mozambique	Initial self-financing (Phase 1)	Countries within gross national income per capital equal to or below US$1085
Nepal	Preparatory transition (Phase 2)	Countries within gross national income per capita between US$1085 and US$1730

*Information collected from Gavi, The Vaccine Alliance Vaccine Application Process Guidelines (2023)* [8]*.*

## 4 Conclusion

Workshops conducted to inform cost of MI delivery studies in five LMICs in Asia and Africa provided an opportunity for key stakeholders to deliberate on needs, programmatic strengths, and opportunities for introducing new maternal vaccines. We found key similarities across countries but also noted important country-specific differences.

Stakeholders widely acknowledged that TT-containing vaccines are currently given safely and effectively during pregnancy and existing infrastructure of countries' health systems provide a template for introducing new maternal vaccines. We found common expectations across countries that de novo systems will not be needed to accommodate new maternal vaccines and a preference for integrating the vaccines within existing immunisation and MCH programs. Bangladesh was the only country that did not acknowledge sub optimal coverage of maternal tetanus immunisation, and participants from all countries cited the need to strengthen current immunisation systems before new vaccines can be delivered successfully. In Bangladesh and Nepal, stakeholders specifically cited the need to overcome health worker shortages to ensure successful introduction of new maternal vaccines.

LMIC health programs are often driven by global priorities. New maternal vaccines were viewed by many participants as an abstract idea far on the horizon, especially practitioners who often confront more immediate priorities. While we found limited knowledge of new maternal vaccines and late-stage candidates and their target diseases among some participants, there was widespread acknowledgement that the workshops provided an early opportunity to raise awareness and inform country readiness and decision-making, well before products are available. All countries identified this as crucial for prioritizing introduction decisions and eventually implementing MI successfully. In September 2024, WHO's Strategic Advisory Group of Experts on Immunisation (SAGE) recommended the introduction of maternal vaccines and/or monoclonal antibodies for the prevention of severe RSV disease in infants in all countries [4]. This recommendation raises the profile of RSV prevention and will likely translate into increased interest in these products, as well as other maternal vaccine candidates, such as those against GBS.

Regardless of the country's stage of Gavi transition to fully self-funding their immunisation program, stakeholders from all countries expressed concern about financial limitations and competing priorities posing significant barriers to new vaccine introduction, including maternal vaccines. A pervasive sentiment was the importance of having a sustainable financing plan, particularly given that countries are facing increased budget constraints and a shrinking fiscal space. Since these products are expected to be cost-effective and, in some countries, may even be cost-saving (for example, see Mahmud et al. 2023 [9]), timely sharing of information (including robust health economic estimates) with policy makers is critical for informed decision making.

An exciting new era is upon us in which new maternal vaccines are becoming available globally. With informed decision-making and effective implementation, their life-saving potential can be reached.

## Credit authorship contribution statement

**Ranju Baral:** Writing – review & editing, Methodology, Formal analysis, Data curation, Conceptualization. **Jessica A. Fleming:** Writing – review & editing, Methodology, Data curation, Conceptualization. **Iracema Barros:** Writing – review & editing, Data curation. **Patience Cofie:** Writing – review & editing, Data curation. **Patience Dapaah:** Writing – review & editing, Data curation. **Sadaf Khan:** Writing – review & editing. **Sophia J. Knudson:** Writing – review & editing. **Sandeep Kumar:** Writing – review & editing, Data curation. **Kamran Mehedi:** Writing – review & editing, Data curation. **Patrick K. Munywoki:** Writing – review & editing, Data curation. **Lauren Newhouse:** Writing – review & editing. **Bryan O. Nyawanda:** Writing – review & editing, Data curation. **Joyce U. Nyiro:** Writing – review & editing, Data curation. **Joseph Odiyo:** Writing – review & editing, Data curation. **Elkanah Otiang:** Writing – review & editing, Data curation. **Rosemond Owusu:** Writing – review & editing, Data curation. **Clint Pecenka:** Writing – review & editing, Methodology, Conceptualization. **Melanie Picolo:** Writing – review & editing, Data curation. **Judite Pinto:** Writing – review & editing, Data curation. **Diana Quelhas:** Writing – review & editing, Data curation. **Satyabrata Routray:** Writing – review & editing, Data curation. **Sarah Sultana:** Writing – review & editing, Data curation. **Surendra Uranw:** Writing – review & editing, Data curation.

## Declaration of competing interest

All authors have no competing interests to declare.

## Data Availability

Data will be made available on request.

## Appendix A Supplementary data

Supplementary material Image 1
