# Supplementary material for: What will it take? Perspectives from five low- and middle-income countries on opportunities and challenges of introducing new maternal vaccines
Source: Vaccine. 2025 Jan 25;45:None. doi: 10.1016/j.vaccine.2024.126654 (PMC11773375; doi:10.1016/j.vaccine.2024.126654)
Supplement: Supplementary file 1 — Supplementary material [file mmc1.docx]

## ***Supplementary material***

### Supplementary material 1: Number and affiliation of country stakeholder participants

|  | Bangladesh | Ghana | Kenya | Mozambique | Nepal |
| --- | --- | --- | --- | --- | --- |
| Ministry of Health and regional/provincial/district health office representatives | 25 | 25 | 19 | 18 | 19 |
| Academic/research organization, National Immunisation Technical Advisory Group (NITAG) members, and professional association representatives | 22 | 8 | 17 | 10 | 11 |
| Non-Governmental Organization representatives | 10 | 15 | 8 | 8 | 7 |
| Participants (#) | 57 | 48 | 44 | 36 | 37 |

### Supplementary material 2: Discussion prompts used during the workshops

| Topics | Discussion questions by thematic area |
| --- | --- |
| I. Procurement, logistics, and supply chain management | |
|  | - How would procurement, logistics, and supply chain management need to adapt for maternal immunization (MI) interventions?   *(Consider procurement; distribution; cold chain; logistics management; waste management; other)* |
|  | - What new or additional procurement, logistics, and supply chain management systems, resources, or procedures would be needed, if any? |
|  | - How would roles and responsibilities work or need to change across EPI and ANC? What would stay the same? |
|  | - What are the biggest opportunities and challenges you foresee around MI intervention procurement, logistics, and supply chain management? |
|  | Additional questions |
|  | - What data do you currently use to quantify maternal tetanus vaccine demand? |
|  | - Will it be necessary to establish an additional distribution channel/system to accommodate new maternal vaccines (like RSV)? At what levels of the health system? |
|  | - How/where are MI vaccines currently stored? |
|  | - Do facilities that provide ANC care have the capacity to store and distribute vaccines? |
|  | - How are logistics management information systems managed? |
|  | - Will it be necessary to establish waste management systems at ANC clinics for new maternal vaccines? |
| II. Program planning and management | |
|  | - How would program planning and management need to adapt for new MI interventions?   *(Consider different levels/divisions of the health system; microplanning; other)* |
|  | - What new or additional program planning and/or management mechanisms would be needed, if any? |
|  | - How would roles, responsibilities, and interactions work or need to change across EPI and ANC programs? |
|  | - What are the biggest opportunities and challenges you foresee around new MI intervention program planning and management? |
|  | Additional questions |
|  | - Are MNCH experts represented in your NITAG or similar committee? |
|  | - What policy adaptations are needed for introduction of MI interventions? |
|  | - How do ANC and EPI programs interact to coordinate MI vaccines at the regional and national levels, and at all operational levels? |
|  | - What major program responsibilities, at various levels of health system, could be shared between ANC and EPI for MI intervention delivery? |
|  | - What kinds of coordination planning activities are needed to introduce MI interventions? At all levels, who needs to be engaged? |
|  | - How do the existing annual ANC and EPI planning/microplanning activities need to be adapted for MI interventions? |
| III. Training and supervision | |
|  | - How would training and supervision need to adapt for MI interventions?   *(Consider different levels of the health system; professional level of training needed; training recipients and providers; training programs/mechanisms needed; other)* |
|  | - What new or additional workforce and supervision training mechanisms would be needed, if any? |
|  | - How would roles, responsibilities, and interactions work or need to change across EPI and ANC programs? |
|  | - What are the biggest opportunities and challenges/barriers you foresee around MI intervention training and supervision? |
|  | Additional questions |
|  | - What is the professional profile of the workforce providing ANC and immunization service delivery? |
|  | - What is the capacity of EPI vaccinators to vaccinate pregnant women during routine visits? |
|  | - What is the capacity of ANC service providers to vaccinate pregnant women during routine ANC visits? |
|  | - In what areas do ANC and EPI staff need additional training for MI interventions? |
|  | - Who needs to be trained on what for MI interventions delivery? |
|  | - How is maternal immunization delivery currently supervised? At different levels of health system? |
| IV. Vaccine administration and surveillance | |
|  | - How would vaccination administration and surveillance need to adapt for MI interventions?   *(Consider vaccination locations, tactics (routine, campaign, etc.), providers, timing; training; roles & responsibilities across EPI and ANC; AEFI tracking; pregnancy outcome tracking; different levels of care and geographies; other)* |
|  | - How would vaccination administration systems need to adapt, if at all? |
|  | - How would surveillance systems need to adapt? |
|  | - What are the biggest opportunities and challenges/barriers you foresee around MI intervention administration and surveillance? |
|  | Additional questions |
|  | - Are there opportunities to vaccinate pregnant women and newborns during existing ANC and EPI visits? |
|  | - What are the main human resource challenges in delivering MI vaccine? |
|  | - For women who receive their tetanus vaccination at the same facility where they receive their ANC, is vaccination available daily or only on scheduled days? |
|  | - How do children born at home get access to birth dose vaccine? |
|  | - What disease surveillance systems are in place for maternal vaccine and other maternal health conditions? |
|  | - Are health providers who vaccinate pregnant women specifically educated on how to report AEFI following vaccination during pregnancy? |
| V. Monitoring and evaluation (Information systems) | |
|  | - How would monitoring & evaluation (M&E) and information systems need to adapt for MI interventions?   *(Consider different levels of care and geographies; vaccination follow up system/reporting tools; missed vaccinee follow up; provider reporting; other)* |
|  | - What new or additional M&E and/or information systems would be needed, if any? |
|  | - How would roles, responsibilities, and interactions work or need to change across EPI and ANC programs at different levels of the health system? |
|  | - What are the biggest opportunities and challenges/barriers you foresee around MI intervention M&E? |
|  | Additional questions |
|  | - What tools and systems are in place currently for monitoring maternal vaccinations? At different levels of health system? |
|  | - Are ANC and EPI both currently included in the national health management information system? |
|  | - How is ANC service routinely monitored? |
|  | - Do you have a system (defaulter tracking) in place to follow up with women who missed scheduled ANC visits or MI (TT) visits? |
|  | - Do you have a system in place to follow up with children born at home for vaccination? |
|  | - What kind of recording and reporting tools need to be developed or modified to meet the needs for MI interventions? |
| VI. Demand creation (sensitization, social mobilization, and communication) | |
|  | - How would demand creation (sensitization, communications, and social mobilization) need to adapt for MI interventions?   *(Consider different levels of the health system or geographies; advocacy & communications strategic planning; priority audiences; key information needs; communications materials; dissemination tactics; community engagement/social mobilization; advocate/communicator training; other)* |
|  | - What new or additional demand generation activities would be needed, if any, given existing TT maternal immunization and birth dose vaccination demand generation strategies? |
|  | - How would roles, responsibilities, and interactions work or need to change across EPI and ANC programs at different levels of the health system? |
|  | - What are the biggest opportunities and challenges/barriers you foresee for MI intervention demand generation? |
|  | Additional questions |
|  | - Are there routine demand creation activities targeted toward MI (TT) and EPI? |
|  | - What communication materials and resources are needed to introduce MI interventions? Consider targeted vaccination (restricted age window for vaccination, seasonal vaccination options). |
|  | - What initial sensitization activities are needed and for whom for MI interventions? At national, regional, and sub-regional levels? |
|  | - What community level social mobilization activities are necessary for the uptake of MI interventions? |
|  | - Which department (at all levels) may be involved / work together for social mobilization activities at community levels? What kind of activities may be needed for the uptake of MI interventions? |
